# Resistance to thyroid hormone beta (R243Q) with autoimmune primary hypothyroidism: report of a kindred

**DOI:** 10.20945/2359-4292-2025-0150

**Published:** 2025-10-06

**Authors:** Alpesh Goyal, Rahul Gupta, Rekha Singh

**Affiliations:** 1 Department of Endocrinology and Metabolism, All India Institute of Medical Sciences, Bhopal

**Keywords:** Thyroid hormone resistance syndrome, Hypothyroidism, Thyroid autoimmunity, Atypical thyroid function tests

## Abstract

The syndrome of resistance to thyroid hormone beta (RTHβ) is characterized by impaired tissue responsiveness to thyroid hormone and manifests as non-suppressed thyrotropin despite elevated thyroid hormone levels. RTHβ is most often caused by missense mutations in the thyroid hormone receptor beta (*THRB*) gene and is typically inherited in an autosomal dominant manner. Autoimmune thyroid disease is the most common cause of primary hypothyroidism worldwide. Association of RTHβ with autoimmune hypothyroidism is extremely rare. We describe an Indian kindred with a similar association. The proband, a 49-year-old female, manifested elevated anti-thyroglobulin antibodies, with an unusual thyroid function test pattern, suggesting mid-normal to high-normal free thyroid hormone levels despite a significantly elevated thyrotropin (free T3 = 4.2 pg/mL; free T4 = 1.37 ng/dL; thyrotropin = 91.82 mIU/L). Family screening revealed a biochemical picture consistent with RTHβ in her elder sister, treated for hypothyroidism, and a daughter who is presently euthyroid with raised anti-thyroglobulin autoantibodies. Both the proband and her daughter harbored a missense mutation in exon 8 of the *THRB* gene (c. 728 G>A; p. Arg243Gln) that is known to impair TRβ function in experimental studies. Considering the hypothyroid state, the proband was started on levothyroxine, with a target to maintain thyrotropin in the normal range, while the daughter received beta-blocker therapy, which relieved her palpitations. To conclude, the coexistence of RTHβ and primary hypothyroidism can delay diagnosis as thyroid hormone levels appear deceptively normal. A discordance between thyrotropin and thyroid hormone, i.e., high thyrotropin with inappropriately normal or high-normal thyroid hormone levels, should prompt this association.

## INTRODUCTION

Resistance to thyroid hormone (RTH), encompassing defects in thyroid hormone (TH) action, is a rare (estimated incidence 1 in 40,000 to 50,000 live births), dominantly inherited disorder characterized by impaired tissue responsiveness to TH (^1-3^). In its more common form (RTHβ), the biochemical hallmark is elevated THs in the face of non-suppressed thyrotropin (TSH) levels. The diagnosis of RTHβ is challenging and requires consideration of several close differentials, including TH-binding globulin (THBG) issues, assay interferences, TSH-producing pituitary adenoma (TSHoma), and other genetic disorders, such as familial dysalbuminemic hyperthyroxinemia (FDH) (^1,4^). Very rarely, primary thyroid dysfunction, in the form of hypo- or hyperthyroidism, may coexist with RTHβ and further challenge the clinical diagnosis (^5-13^). We present the report of a genetically proven RTHβ kindred, wherein coexistent autoimmune hypothyroidism in the proband created diagnostic delays and dilemmas. 

## CASE REPORT

A 49-year-old female presented to our outpatient department in January 2024 for evaluation of a thyroid problem that remained “unsolved” over the years (Table 1). Back in August 2013, she was evaluated with a thyroid function test (TFT) for weight gain and mild behavioral problems, revealing high THs (T3 = 186 ng/dL [N: 60-200 ng/dL]; T4 = 17.1 µg/dL [N: 4.5-12.0 µg/dL]) with unsuppressed TSH (4.53 mIU/L [N: 0.3-5.5 mIU/L]). No specific treatment was initiated, and TSH surveillance was done over the next 6 to 7 years, with values ranging between 2.39 and 9.71 mIU/L. In January 2020, she was started on levothyroxine (LT4) therapy, considering the trend of persistent TSH elevation. However, after an initial normalization, TSH was found to increase again (6.95 to 10.47 mIU/L), and TH levels persisted in the high range (T3 = 179 to 199 ng/dL; T4 = 17.3-19.0 µg/dL), despite an adequately compliant LT4 dose of 100 µg per day. She was advised to stop treatment in July 2022, and a repeat TFT performed at an interval of 8 weeks revealed severely elevated TSH (91.82 µIU/mL) with mid-normal free T4 (1.37 ng/dL) and high-normal free T3 (4.37 pg/mL) levels. At this time, she restarted LT4, but an abnormal TFT trend with high THs and unsuppressed TSH persisted. She did not use estrogen or oral contraceptive pills, and her free hormone levels were elevated on more than one occasion.

**Table 1:** Serial thyroid function tests of the proband

Time	T3 (ng/dL)	T4 ( µg/dL)	FT3 (pg/mL)	FT4 (ng/dL)	TSH (mIU/L)	Remarks
2013 Aug^a^	186	17.1	-	-	4.53	Index TFT
2020 Jan^b^	-	-	-	-	8.61	Started LT4
2020 July^b^	162	17.7	-	-	3.74	Ongoing LT4
2022 July^b^	179	17.3	-	-	6.95	Ongoing LT4
2022 July^c^	199	19.0	-	-	10.47	Ongoing LT4
2022 Sept^b^	-	-	4.27	1.37	91.82	After stopping LT4 for 8 weeks
2022 Nov^b^	240	24.4	-	-	10.5	Ongoing LT4
2023 Feb^b^	-	-	5.59	2.35	5.58	Ongoing LT4
2024 Jan^d^	195	17.31	-	1.62	4.94	Ongoing LT4
2024 Jan^e^	182	18.0	-	2.64	4.45	Ongoing LT4

Reference ranges: ^a^ T3 = 60-200 ng/dL; T4 = 4.5-12.0 µg/dL; TSH = 0.3-5.5 mIU/L;

b T3 = 58-159 ng/dL; T4 = 4.5-12.0 µg/dL; FT3 = 1.7-4.2 pg/mL; FT4 = 0.7-1.8 ng/dL; TSH = 0.35-4.94 mIU/L;

c T3 = 60-181 ng/dL; T4 = 5.01-12.45 µg/dL; TSH = 0.35-5.5 mIU/L;

d T3 = 70-193 ng/dL; T4 = 5-12 µg/dL; FT4 = 0.7-1.48 ng/dL; TSH = 0.35-4.94 mIU/L;

e T3 = 80-200 ng/dL; T4 = 5.1-14.1 µg/dL; FT4 = 0.93-1.78 ng/dL; TSH = 0.27-4.2 mIU/L. TSH: thyrotropin.

**Figure 1:**
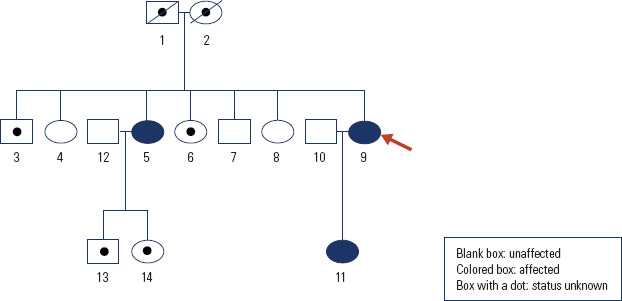
Pedigree chart of the reported kindred (proband is indicated by an arrow).

At the time of presentation to our department, she received 750 µg per week LT4 (100 µg for 5 days and 125 µg for 2 days). There were no symptoms referable to thyroid dysfunction. She had a history of systemic hypertension for the past 3 years, adequately controlled on clinidipine (10 mg per day) and telmisartan (40 mg per day). She had regular menstrual cycles, and her obstetric history suggested first-trimester miscarriage in the first pregnancy and a healthy daughter (no. 11 on the pedigree chart; Figure 1) born of the second pregnancy. She (no. 9 on the pedigree chart) was the youngest of the seven siblings, and her elder sister (no. 5 on the pedigree chart) was also treated for hypothyroidism elsewhere. Examination revealed a middle-aged female with a height of 152.5 cm, weight of 58.9 kg, and body mass index of 24.9 kg/m^2^. Blood pressure was 150/84 mmHg, and heart rate was 78 beats per minute, with regular rhythm. There was soft, grade 1 goiter, but no hand tremors, signs of thyroid orbitopathy or dermopathy, or features of other autoimmune disorders. The rest general and systemic examination was unremarkable.

Her blood parameters, including complete blood count, liver and renal function tests, calcium profile, and fasting plasma glucose and glycated hemoglobin, were normal. We repeated her TFT from two other automated immunoassay platforms – Abbott Architect (Chemiluminescent Microparticle Immunoassay or CMIA), and Roche Elecsys (Electrochemiluminescent Immunoassay or ECLIA) – to exclude assay interferences. Both the platforms revealed a consistent biochemistry of elevated THs with unsuppressed TSH (January 2024; Table 1). At this time, LT4 was decided to be discontinued, and a TFT was repeated after a 4-week interval. There was an increase in TSH to 12 mIU/L (n = 0.35-4.94), but TH levels declined only marginally, remaining at or above the upper limit of normal (T3 = 180 ng/dL [n = 70-193 ng/dL]; T4 = 15.2 µg/dL [n = 5-12 µg/dL]; free T4 = 1.56 ng/dL [n = 0.7-1.48 ng/dL]).

### Differential diagnosis and further investigative work-up

Differential diagnoses of high THs with inappropriate normal or elevated TSH (referred as discrepant or atypical TFT) include: THBG excess, either inherited or acquired, acute non-thyroidal illness (NTI), use of drugs such as amiodarone and heparin, FDH caused by albumin (*ALB*) gene mutations, RTHβ due to TH receptor β (*THRB*) gene mutations, TSHoma, assay interference due to TH autoantibodies and heterophile antibodies causing false elevation of THs and TSH, respectively, TH transporter defect due to monocarboxylate transporter 8 (*MCT8*) gene mutations, TH metabolism defect due to selenocysteine insertion sequence binding protein 2 (*SBP2*) gene mutations, and poorly compliant LT4 therapy with intermittent dosing prior to blood testing (^1,14-16^). As discussed, the patient had elevated free TH levels (excluding TBG excess), there was no clinical setting for acute NTI or use of offending medications, both T3 and T4 were uniformly elevated (generally isolated T4 elevation is noted in FDH), consistent biochemistry was noted on different assay platforms (rendering TH autoantibody and heterophile antibody-related assay interference less likely) and the abnormal TFT pattern preceded LT4 initiation (excluding poor LT4 compliance as a possibility). Patients with MCT8 mutations have high T3, low T4, and psychomotor retardation, while those with SBP2 mutations have high T4, low T3, and growth delay, which were not present in the index case (^17,18^). Thus, the two main differentials entertained at this time were RTHβ and TSHoma. Besides, a decreased thyroidal reserve due to underlying primary hypothyroidism was also considered, given an elevated serum TSH level (>6 mIU/L) (^1^).

Next, we performed a family screening, which revealed a similar biochemical phenotype in her daughter (no.11; T3 = 184 ng/dL, T4 = 16.6 µg/dL, TSH = 3.08 mIU/L) and elder sister (No. 5; T3 = 213 ng/dL, T4 = 15.7 µg/dL, TSH = 6.65 mIU/L; on LT4 = 100 µg per day) and a normal TFT pattern in other family members (nos. 4, 7 and 8) (Figure 1 and Supplementary Table 1). The daughter, aged 20 years, was healthy, had a normal birth and developmental history and academic performance, but complained of palpitations since childhood. Examination revealed a heart rate of 100 beats per minute, soft, grade 1 goiter, but no other features of hypo- or hyperthyroidism. This favored a possibility of RTHβ, and sex hormone binding globulin (SHBG) was measured as a biomarker of TH action in both the proband and her daughter at baseline. Both had normal SHBG levels (33 and 61 nmol/L, respectively; n = 10.8-180 nmol/L) in the face of increased THs, supporting resistance to TH action at the liver via TRβ.

Prior to the current presentation, the proband had a pituitary MRI done, which was normal, and her pituitary hormone profile was unremarkable. We did not have access to TSH α-subunit assay or thyrotropin releasing hormone (TRH), and proceeded next to T3 suppression test in the proband (LT3 = 25 µg twice daily on days 1 to 3, 50 µg twice daily on days 4 to 6, and 75 µg twice daily on days 7 to 9). In line with RTHβ, the TSH response to T3 was present but attenuated (TSH declined from 12.82 mIU/L at baseline to 0.16 mIU/L at day 10, but did not suppress completely) (^1,19^). Results of changes in several parameters measured during the T3 suppression test are provided in Table 2.

**Table 2:** Results of T3 suppression test in the proband

Parameter	Baseline	Day 10	Change (%)
Weight, kg	56.7	57	+0.5
Sleeping HR, per minute	79	97	+22.8
T3, ng/dL	219	> 600	+>174
T4, µg/dL	18.82	7.12	-62.2
FT4, ng/dL	1.76	0.85	-51.7
TSH, mIU/L	12.82	0.16	-98.8
Cholesterol, mg/dL	234	133	-43.2
LDL-cholesterol, mg/dL	151	76	-49.7
CPK, IU/L	144.4	51.2	-64.5%
ALP, IU/L	77.2	105.9	+37.2%

HR: heart rate; T4: thyroxine; TSH: thyrotropin; LDL: low density lipoprotein; CPK: creatine phosphokinas; ALP: alkaline phosphatase.

**Figure 2:**
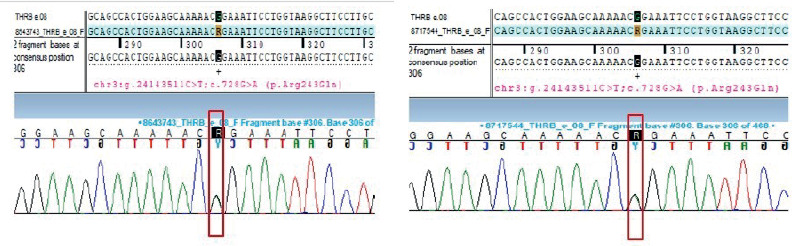
Electropherogram showing heterozygous missense variation (c.728 G>A) in the *THRB* gene in the proband (left panel) and her daughter (right panel).

Finally, next-generation sequencing (NGS) was performed in the proband, which revealed a pathogenic heterozygous missense variant (c. 728 G>A; depth 97x) in exon 8 of the *THRB gene*, resulting in substitution of arginine by glutamine at codon 243 (p. Arg243Gln or R243Q). This variant is rare, is predicted to be damaging or probably damaging by computational algorithms, and the affected codon is conserved across species. Furthermore, the variant has been previously reported in 20 families with RTHβ and impairs protein function in experimental studies (^1,20,21^). Sanger sequencing further validated the NGS findings and also confirmed the presence of the same variant in her affected daughter, indicating familial segregation (Figure 2). A clinical visit and genetic confirmation for the affected elder sister are awaited at the time of writing this report.

Etiological work-up of hypothyroidism in the proband revealed negative thyroid peroxidase antibodies but raised anti-thyroglobulin antibodies (> 1,000 IU/mL; n < 4 IU/mL) and ultrasonographic evidence of thyroiditis. Similarly, the euthyroid daughter presented negative thyroid peroxidase and elevated anti-thyroglobulin antibodies (22 IU/mL).

### Management and follow-up

The family was counseled regarding the benign nature of the condition. Given the evidence for reduced intra-thyroidal reserve in the proband, LT4 75 µg once daily was initiated (TFT at initiation: T3 = 154 ng/dL, T4 = 13 µg/dL, TSH = 8.9 mIU/L), with a target to maintain normal TSH and T4 levels in the baseline range (around 17 to 18 µg/dL). These high levels of T4 were chosen as a target, since the HPT axis set point is altered in patients with RTHβ, such that higher than normal TH levels are required to maintain normal TSH (these levels are reflected in the index TFT). After 4 months, the patient was asymptomatic, her T3 remained stable at 159 ng/dL, T4 increased to 18.6 µg/dL, and TSH declined to 4.3 mIU/L, and the same dose was maintained, with a plan for TFT monitoring at every 3 to 6-month interval. For the daughter, propranolol long-acting preparation (40 mg per day) was started, with relief in palpitations (her heart rate reduced to 70 to 80 beats per minute), and a plan is in place to monitor TFTs every 6 to 12 months for evolution of primary hypothyroidism.

## DISCUSSION

RTHβ was first described in 1967 by Samuel Refetoff and cols. as a familial syndrome manifesting as deaf-mutism, dysgenetic stippled epiphysis, goiter, and high protein-bound iodine (PBI) (^22^). However, the molecular basis was only unveiled in 1989, when *THRB* gene mutations were identified to cause this clinical syndrome (^23^). In RTHβ, the mutant receptor exerts a dominant negative effect, implying that it not only has reduced function, but also interferes with the function of wild-type TRβ. More than 600 families of RTHβ have been identified till date (^1^), however, the coexistence of RTHβ with primary thyroid dysfunction is rare (^5-13^).

In the current proband, RTHβ was suspected due to biochemical phenotype of high TH levels with unsuppressed TSH, in the absence of interfering drugs, NTI, and binding protein issues. A positive family history, normal SHBG levels despite elevated THs, normal pituitary imaging, and partial TSH suppression (to < 1 mIU/L) on supraphysiological T3 dosing supported the diagnosis (^1-3,19^). The diagnosis was finally confirmed through molecular testing. Approximately 85% of cases of RTHβ have a germline mutation in the *THRB* gene; more than 200 mutations have been described, and most are missense in nature. These mutations affect the T3-binding or hinge region of the TRβ protein and are distributed across three clusters spanning exons 7-10 of the *THRB* gene (cluster 1: 426-460 codons; cluster 2: 302-357 codons; cluster 3: 234-282 codons) (^1^). The proband and her daughter harbored codon 243 (R243Q; cluster 3) mutation in exon 8 of the *THRB* gene, which is known to affect the hinge region of the protein and impact T3 action. Unlike mutations in the ligand-binding domain of TRβ, the R243Q mutation does not affect T3-binding affinity *in vitro*; however, it markedly impairs the ability of T3 to dissociate the mutant receptor homodimer or release the corepressor from the thyroid response element (^20,21^). Of note, other cluster 3 mutations, such as V264D and T277A, are associated with reduced T3-binding affinity as well as impaired T3-dependent release of corepressors (V264D) and recruitment of coactivators (T277A) (^24,25^).

The onset of primary thyroid dysfunction, manifesting as rising TSH levels, complicated the clinical picture in the proband. At any level of TSH, patients with RTHβ have higher than expected circulating TH levels; in the proband, when TSH increased to >90 mIU/L, free T4 remained in mid-normal range, rather than declining to frankly low levels. Thus, the correlation between free T4 and TSH is disturbed at every stage in RTHβ. Another tool, Thyrotroph T4 Resistance Index (TTRI), calculated as the product of free T4 (% upper limit of normal or ULN) and TSH (mIU/L), provides a useful estimate of thyrotroph sensitivity to THs and disease severity (^1,21^). In a study of 23 patients with RTHβ and 26 healthy controls, TTRI levels were significantly higher in patients with RTHβ (664 ± 231 in patients with R243Q/R243W mutations and 308 ± 138 in patients with R320H mutations), compared to controls (136 ± 73) (^21^). In our proband (R243Q), TTRI levels were 6988 [free T4 = 1.37 ng/dL (76.1% ULN) and TSH = 91.82 mIU/L], when she was grossly hypothyroid, and varied between 541 and 660 [free T4 = 1.62 ng/dL (109.5% ULN) and TSH = 4.94 mIU/L; free T4 = 2.64 ng/dL (148.3% ULN) and TSH = 4.45 mIU/L), when a relative euthyroid state was maintained on LT4 therapy.

Etiological evaluation of hypothyroidism revealed negative thyroid peroxidase but elevated anti-thyroglobulin antibodies in the proband and her daughter; this combination is described in about 5% of patients with chronic autoimmune thyroiditis (^26^). Thus, autoimmune thyroid disease coexisted in our kindred; the proband (and possibly her elder sister) manifested primary autoimmune hypothyroidism, while her daughter is presently euthyroid. The association between RTHβ and thyroid autoimmunity was initially considered coincidental. However, a 2010 study by Barkoff and cols. found otherwise (^27^). The authors evaluated 330 genetically confirmed RTHβ individuals and 92 unaffected first-degree relatives and reported 2.36-fold higher odds of thyroid autoantibodies in individuals with RTHβ compared to their unaffected relatives (^27^). The proposed mechanisms include stimulation of the immune system by high THs via thyroid receptor alpha, and release of pro-inflammatory cytokine TNF-alpha by the lymphocytes under the stimulatory effect of chronic TSH elevation (^3,10,27-30^). Similar associations of autoimmune hypothyroidism or hyperthyroidism (Graves’ disease) with RTHβ have been rarely described in the literature, with the coexistence suspected when free T4 levels were normal despite a grossly elevated TSH (as in our case), or high TH levels persisted despite normalization of TSH with antithyroid drug treatment (contrasting to a routine scenario where recovery of TSH lags behind THs in hyperthyroidism) (^7-13^).

Women with RTHβ have normal fertility; however, when they are pregnant with an unaffected fetus, an increased tendency for miscarriage (three to four fold increased risk), low birth weight, and postnatal TSH suppression has been reported (^3,31^). These adverse events are explained by exposure to a catabolic intrauterine environment caused by high TH levels; there is a 50% chance in every pregnancy that the fetus manifests wild-type TRβ and is susceptible to these adverse effects. In our kindred, the first pregnancy of the proband ended in a miscarriage, and it is possible that the aborted fetus had wild-type TRβ, while the second pregnancy resulted in the delivery of a normal-weight girl child who was later confirmed to have mutant TRβ. Of note, prenatal diagnosis followed by administration of antithyroid drugs beginning in the early second trimester (to RTHβ mothers carrying unaffected fetuses) and targeting free T4 levels in high-normal range (close to 120% ULN), is a prudent strategy associated with normal birth weight and TSH levels, comparable to infants with mutant TRβ (^32^). In the context of our kindred, the daughter (no. 11) needs a close follow-up for the evolution of hypothyroid state, and should she become pregnant in later life, prenatal diagnosis and potential treatment adjustments would warrant consideration.

To the best of our knowledge, this is the second report of a *THRB* gene mutation from India, the previous one being a de novo missense variant (P453S) described in a 6.5-year-old boy (^33^); other phenotypic descriptions have been limited by the absence of genetic diagnosis (^34,35^). We acknowledge certain limitations. First, the elder sister (no.5) had a biochemical phenotype of RTHβ, but genetic confirmation was not available; hence, conclusions cannot be drawn about her disease state. Genetic testing also carries implications for her children (nos. 13 and 14). Second, we attributed the first-trimester miscarriage in the proband to an “unaffected fetus”; however, in the absence of supporting genetic evidence, this observation remains hypothetical. Finally, two of the proband’s first-degree relatives (nos. 3 and 6) refused biochemical testing, precluding complete familial segregation.

Our report highlights an extremely rare presentation of RTHβ, with autoimmune primary hypothyroidism complicating the clinical picture in the proband. In the setting of RTHβ, a TSH level > 6 µIU/mL should prompt suspicion of underlying primary thyroid dysfunction. When RTHβ is complicated with primary hypothyroidism, the mismatch between TH and TSH persists; TH levels may not be frankly elevated but are still inappropriately high for the degree of TSH elevation.

## Figures and Tables

**Supplementary Table 1:** Thyroid function tests of the screened first-degree relatives of the proband

Parameter	No. 4 (elder sister)	No. 7 (elder brother)	No.8 (elder sister)	No. 5 (elder sister)*	No. 11 (daughter)*
T3 (ng/dL)	103	132	111	213*	184*
T4 (µg/dL)	10.32	8.26	9.0	15.7*	16.6*
TSH (mIU/L)	3.74	2.37	1.56	6.65*	3.08*
Comment	No medication	No medication	No medication	LT4 100 µg OD	No medication

The parameters signed with * are abnormal and segregate with the phenotype in proband.

## Data Availability

datasets related to this article will be available upon request to the corresponding author.
